# A Case of Thyroid Carcinoma Combined With Thyroid Hemiagenesis

**DOI:** 10.1155/crie/6636902

**Published:** 2026-02-03

**Authors:** Shaohua Chen, Fengwei Wang, Shenli Huang, Shuting Qin, Junyang Mo, Minmin Zhang, Yan Chen

**Affiliations:** ^1^ Department of Breast and Thyroid Surgery, Liuzhou People’s Hospital Affiliated to Guangxi Medical University, Liuzhou, China; ^2^ Health Management Centre, Liuzhou People’s Hospital Affiliated to Guangxi Medical University, Liuzhou, China

**Keywords:** central neck dissection, differentiated thyroid carcinoma, thyroid carcinoma, thyroid hemiagenesis

## Abstract

**Introduction and Importance:**

Thyroid hemiagenesis (THA) is a rare congenital anomaly characterized by the underdevelopment or complete absence of one thyroid lobe. The coexistence of thyroid carcinoma in patients with this condition is exceedingly uncommon, with only a limited number of cases reported worldwide. Awareness of this presentation is essential due to its diagnostic and therapeutic implications.

**Case Presentation:**

We report a 36‐year‐old female who presented with a right thyroid nodule and was subsequently diagnosed with papillary thyroid carcinoma in the context of left THA. Ultrasonography and contrast‐enhanced computed tomography (CT) confirmed the absence of the left thyroid lobe and isthmus. Fine‐needle aspiration biopsy (FNAB) was not performed due to the small size of the nodule and patient preference. The patient underwent right thyroidectomy with prophylactic right central neck dissection. Intraoperative frozen section confirmed papillary carcinoma, guiding the surgical extent. Postoperative pathology revealed a 0.4 cm papillary carcinoma without lymph node metastasis.

**Clinical Discussion:**

THA is typically asymptomatic and often discovered incidentally. However, anatomical variations may complicate the diagnosis and surgical management of thyroid carcinoma. Preservation of parathyroid glands, recurrent laryngeal nerves, and awareness of possible ectopic thyroid tissue, are critical during surgery.

**Conclusion:**

Papillary thyroid carcinoma occurring in THA is rare but clinically significant. This case highlights the importance of careful preoperative assessment and the value of intraoperative frozen section analysis in determining the need for central neck dissection. Early detection, individualized surgical planning, and multidisciplinary follow‐up can optimize outcomes in such atypical presentations.

## 1. Introduction

Thyroid hemiagenesis (THA) is a rare congenital disorder, with a higher incidence in females, occurring at a rate of ~0.05% to 0.5% [1]. Patients with THA usually remain asymptomatic, and they are often detected incidentally during screening [2]. The contralateral thyroid in these patients may be normal or may show compensatory hypertrophy or hyperplasia, but the risk of developing pathologies increases [3], including thyroiditis, nodular goiter, and thyroid carcinoma. Thyroid carcinoma most commonly arises from the thyroid gland itself; however, it may also originate from ectopic thyroid tissue. Ectopic thyroid tissue results from abnormal migration of the thyroid primordium during embryological development and can be found in various locations along the thyroglossal duct tract, including the lingual, subhyoid, and mediastinal regions. Although uncommon, papillary thyroid carcinoma has been reported to occur in ectopic thyroid tissue, particularly within thyroglossal duct cysts [4]. These rare presentations highlight the complexity of thyroid embryogenesis and emphasize the importance of considering ectopic thyroid carcinoma in differential diagnoses involving cervical masses. This background further underscores the clinical significance of reporting uncommon thyroid carcinoma presentations such as the current case.

## 2. Case Presentation

On April 17, 2023, a 36‐year‐old female presented to Liuzhou People’s Hospital, Guangxi, China, with a complaint of having discovered a right thyroid nodule 11 days earlier. Thyroid function tests were within normal limits (thyroid‐stimulating hormone [TSH]: 2.415 mIU/L, reference range 0.35–4.94 mIU/L; triiodothyronine [T3]: 1.48 nmol/L, reference range 0.98–2.33 nmol/L; thyroxine [T4]: 100.81 nmol/L, reference range 62.88–150.8 nmol/L), and parathyroid hormone levels were also normal.

**Figure 1:**
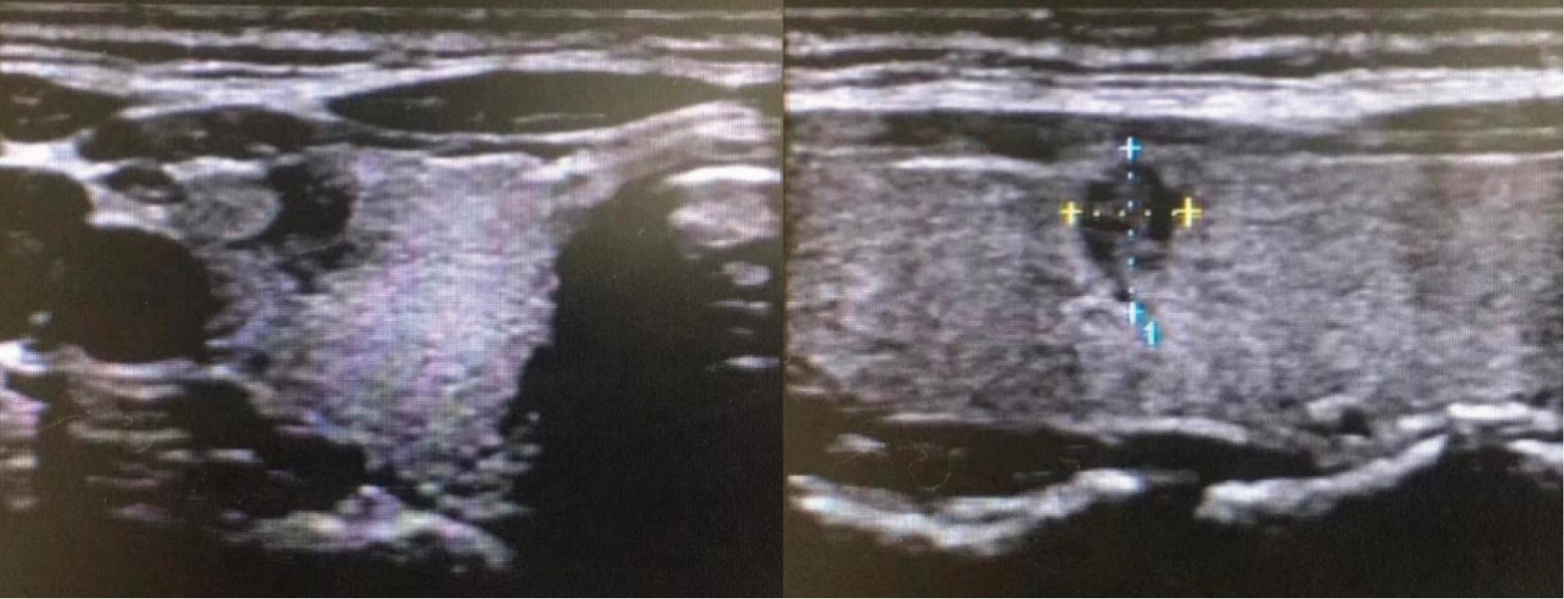
Ultrasound images of thyroid nodules.

**Figure 2:**
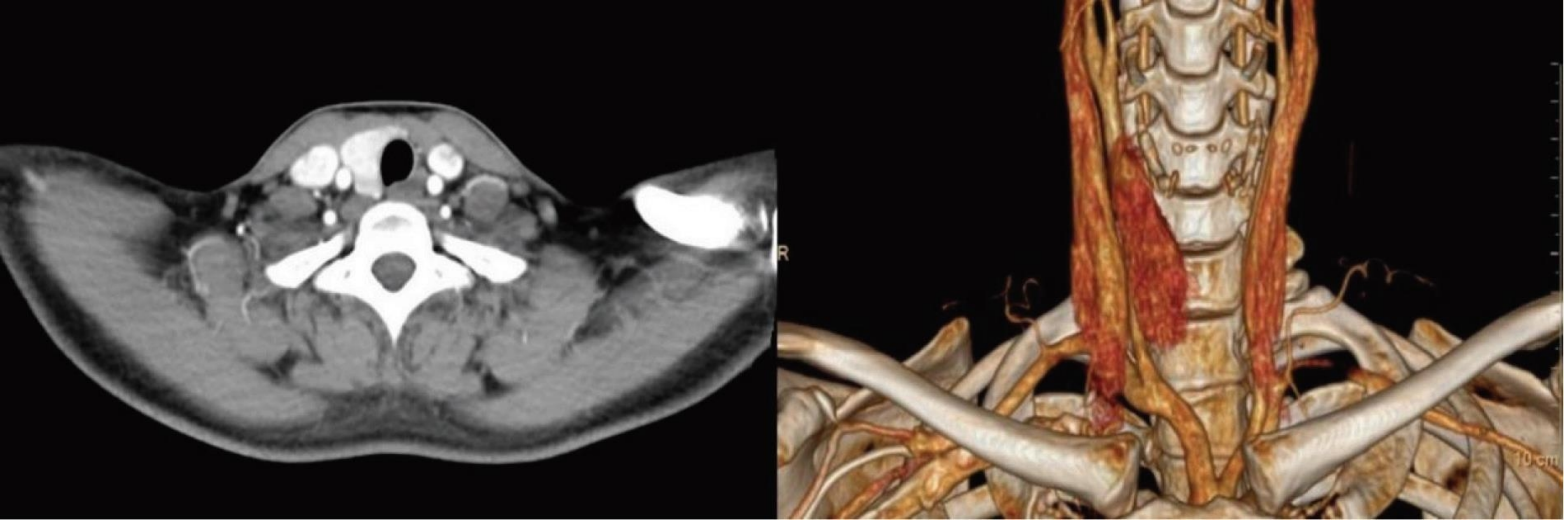
CT and 3D reconstruction of the neck.

Thyroid ultrasonography revealed the absence of the left thyroid lobe and isthmus. The right lobe parenchyma appeared normal, with a solitary nodule detected in the right thyroid lobe (Figure 1). Neck contrast‐enhanced computed tomography (CT) further confirmed the absence of the left thyroid lobe and thyroid isthmus (Figure 2). The patient had no history of thyroid or neck surgery. Based on these findings, the lesion was considered highly suspicious for right‐sided papillary thyroid carcinoma in the setting of left THA.

Although the thyroid nodule was small, with a maximum diameter of 7 mm, preoperative ultrasonography characterized it with the following a TI‐RADS parameters: composition (solid), echogenicity (hypoechoic), shape (wider‐than‐tall), margin (irregular with angular edge), and echogenic foci (microcalcifications). Based on these features, the nodule was classified as TI‐RADS category 5. According to the National Guidelines for the Diagnosis and Treatment of Thyroid Cancer 2022 in China, lobectomy with prophylactic ipsilateral central neck dissection is recommended for differentiated thyroid carcinoma (DTC) <1 cm without high‐risk features. Fine‐needle aspiration biopsy (FNAB) was not performed due to the small size of the nodule and the associated high false‐negative rate. After being informed of these limitations, the patient declined FNAB and elected to proceed with surgical treatment.

**Figure 3:**
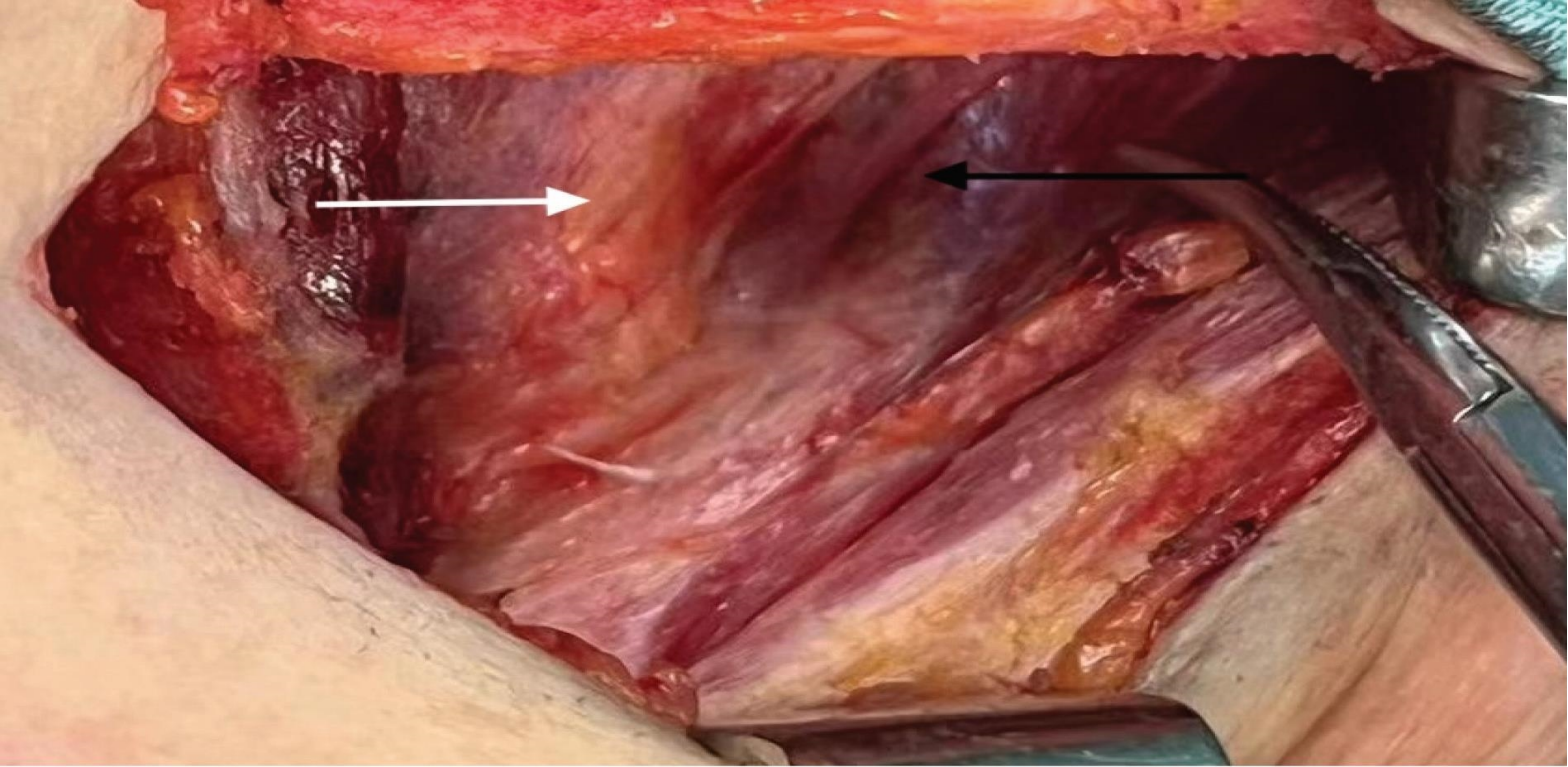
Schematic diagram of intraoperative situation. White arrow shows trachea, black arrow shows left thyroid bed no left thyroid is seen.

Right thyroidectomy with right central neck lymph node dissection was performed, confirming the absence of the left thyroid lobe and presence of the left thyroid artery intraoperatively (Figure 3). Postoperative pathology revealed papillary carcinoma of the right thyroid lobe, with a diameter of 0.4 cm (Figure 4). No lymph node metastasis was observed in the right central neck region (0/3). Final pathological examination confirmed papillary thyroid carcinoma of the right lobe. According to the American Joint Committee on Cancer (AJCC) 8th edition staging system, the tumor was staged as T1aN0M0. Given that the patient was younger than 55 years old, the overall stage corresponds to Stage I disease. At 2 years of follow‐up, there was no recurrence, and the patient was receiving treatment with levothyroxine tablets for the left thyroid absence.

Figure 4Histopathological examination of the right thyroid lobe shows papillary thyroid carcinoma with a tumor size of approximately 0.4 cm. No vascular invasion, nerve infiltration, or capsule involvement is observed. Adjacent thyroid tissue shows nodular goiter changes. (A, 100x): Low‐power view showing complex papillary formations with fibroblastic stromal reaction and fibrosis. (B, 100x): Another low‐power field demonstrating infiltrative growth of tumor cell clusters into the surrounding thyroid parenchyma. (C, 200x): Intermediate‐power view revealing crowded and overlapping tumor cells with loss of nuclear polarity. The nuclei are enlarged and hyperchromatic, with an increased nucleocytoplasmic ratio and nuclear pleomorphism. (D, 400x×): High‐power view highlighting the characteristic nuclear features of papillary thyroid carcinoma, including ground‐glass (orphan Annie–eye–like) nuclei, intranuclear pseudoinclusions, nuclear grooves, nuclear overlap, and irregular nuclear contours.

**Figure:**
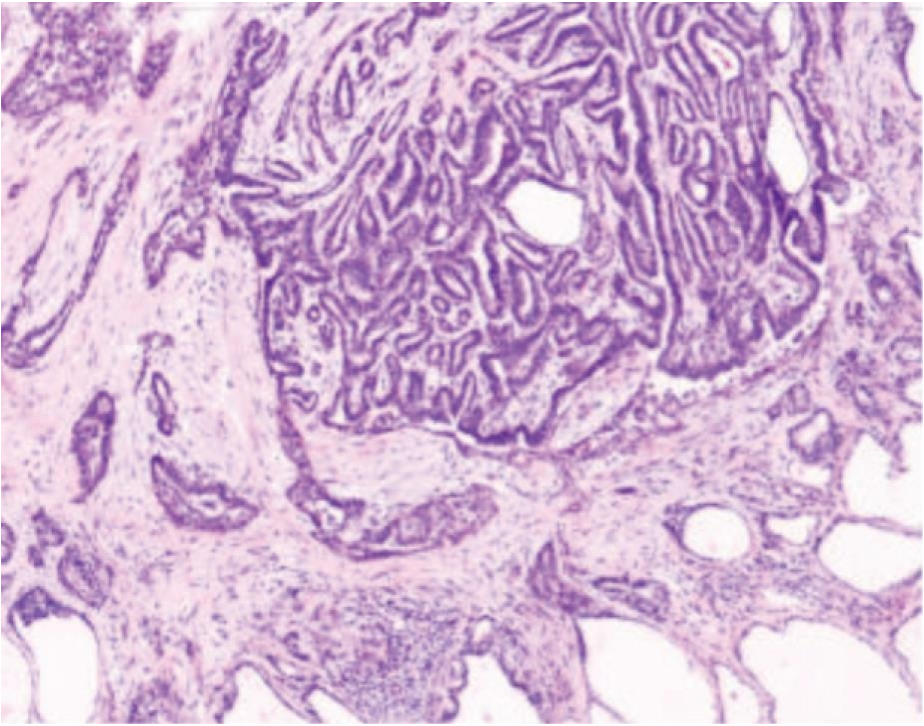
(A)

**Figure:**
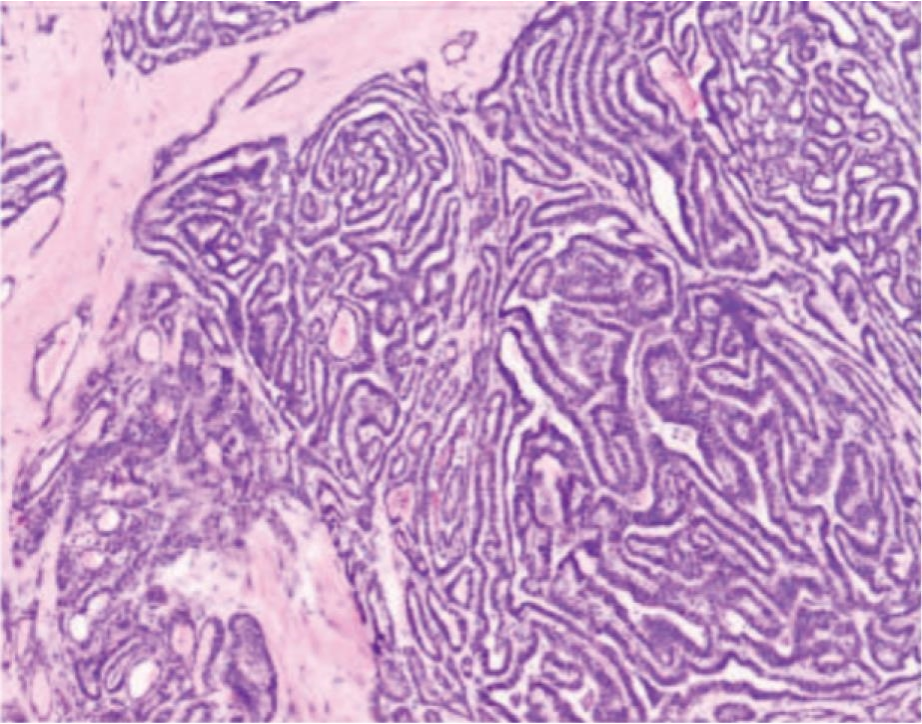
(B)

**Figure:**
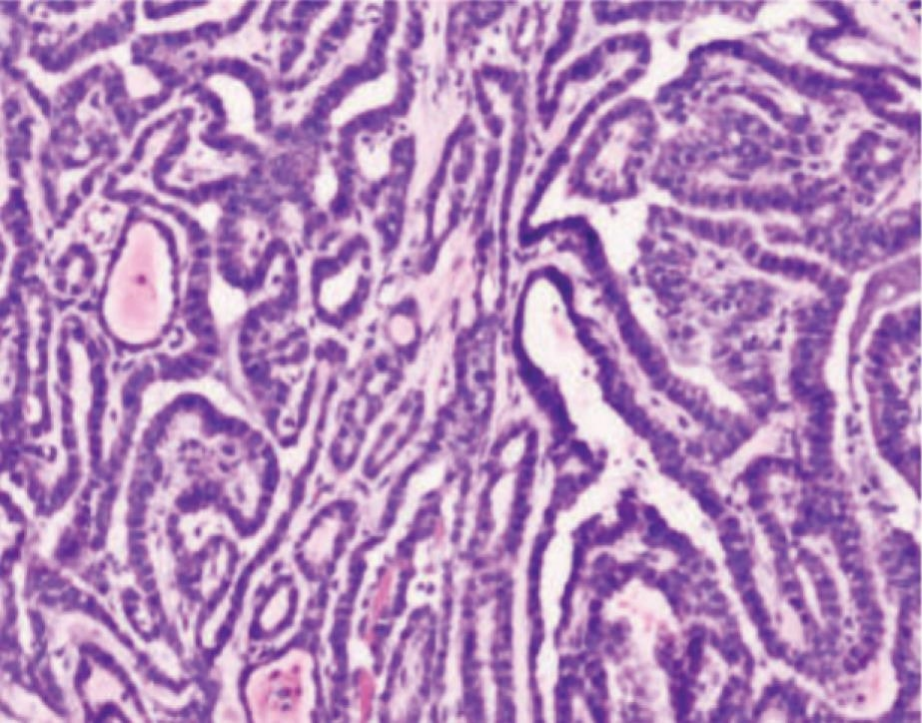
(C)

**Figure:**
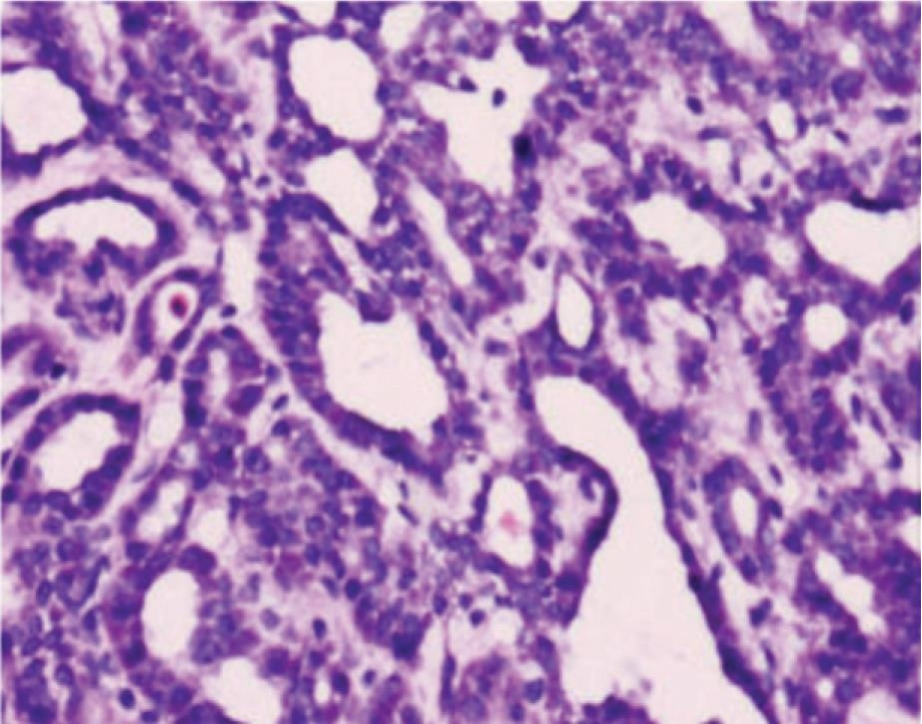
(D)

## 3. Discussion

THA is a rare congenital thyroid disorder characterized by the absence of one thyroid lobe. The most typical presentation involves the absence of the left lobe, accounting for 80% of THA cases [5], while the absence of the isthmus is not consistent [6]. Currently, it is observed that despite the absence of one thyroid lobe in THA, the thyroid function of the contralateral lobe is usually normal, in most cases [2, 7]. However, some studies suggest that in some THA patients with abnormal thyroid function, elevated TSH levels may lead to the development of thyroid carcinoma [8]. Ruchala et al. [9] found that THA patients tend to have higher TSH levels compared to patients with bilateral thyroid glands. Moreover, as time progresses, THA patients exposed to prolonged TSH stimulation have an increased risk of developing other thyroid diseases [10]. Therefore, early intervention should be considered clinically for identified THA patients. In the present case report, a THA patient presented with a thyroid nodule on the right side, normal thyroid function, and a high suspicion of thyroid carcinoma, prompting surgical treatment. Several reports have documented the coexistence of THA and PTC, confirming that although rare, this association has been consistently observed in clinical practice [11–13].Therefore, a thorough understanding of normal thyroid anatomy, its congenital variations, and related pathological changes is essential to ensure surgical safety. Our findings are in accordance with previous reports, particularly regarding the coexistence of THA and PTC and the surgical challenges posed by anatomical variation.

### 3.1. Diagnosis of THA Combined With Thyroid Carcinoma

Currently, ultrasonography is the preferred method for diagnosing thyroid nodules and has a high sensitivity for detecting missing nodules [14]. Due to the convenient and inexpensive nature of ultrasound, Mikosch et al. [15] identified 16 THA patients out of 71,500 patients using ultrasound. Thyroid scintigraphy can also be used for the diagnosis of THA, and in cases of absence of the isthmus thyroid gland, scintigraphy may show a unique hockey stick sign [6]. For the diagnosis of thyroid carcinoma, the same clinical methods currently used in routine practice were applied. In this case, thyroid scintigraphy was not performed due to cost and accessibility considerations. However, contrast‐enhanced CT of the neck and chest revealed no ectopic thyroid tissue in common locations such as the lingual, mediastinal, or retrosternal regions. This constitutes a limitation in confirming the diagnosis of THA, as scintigraphy can help distinguish true hemiagenesis from thyroid atrophy or postsurgical absence.

Thyroid scintigraphy using technetium‐99 m or iodine isotopes is one of the most important diagnostic methods for identifying ectopic thyroid tissue. It can not only demonstrate the presence or absence of thyroid tissue in the normal anatomical location but also detect thyroid tissue distributed in other regions. This examination has high sensitivity and specificity in differentiating ectopic thyroid tissue from other causes of midline neck masses and plays an essential role in the diagnostic evaluation of THA [16].

Furthermore, ultrasonography and CT imaging are valuable for anatomical assessment but cannot fully exclude the presence of functional ectopic thyroid tissue, particularly in patients with normal thyroid function tests [17]. CT and MRI are also essential for preoperative anatomical evaluation and surgical planning in thyroid diseases [18].

In our patient, the diagnosis of THA was established based on the absence of the left lobe and isthmus on both ultrasound and CT, together with intraoperative confirmation. Nevertheless, the absence of scintigraphic evaluation remains a limitation that should be acknowledged when interpreting this case.

### 3.2. Treatment of THA Combined With Thyroid Carcinoma

There is no specific surgical treatment method for THA combined with thyroid carcinoma, and treatment is carried out using conventional methods for thyroid carcinoma [19, 20]. Thyroid carcinomas frequently metastasize to regional cervical lymph nodes, and understanding the anatomical distribution of these nodes is crucial for appropriate surgical planning. The head and neck region contains nearly 300 lymph nodes, categorized into superficial and deep groups, with the deep cervical nodes further subdivided into upper, middle, and lower regions along the internal jugular chain. Among these, the central compartment—which includes the pretracheal, paratracheal, and prelaryngeal lymph nodes—is recognized as the most common basin for early metastasis from papillary thyroid carcinoma [21]. Therefore, even in patients without clinically evident lymphadenopathy, occult metastasis may still be present, and central neck dissection may help improve staging accuracy and reduce recurrence risk when intraoperative findings suggest malignancy. This anatomical and pathological background supports the rationale for prophylactic central neck dissection in selected cases, particularly when thyroid carcinoma coexists with congenital thyroid anomalies such as THA. In addition to the oncologic considerations, the surgical complexity of such cases is further increased by the anatomical abnormalities associated with THA. However, some studies indicate that the anatomical abnormalities in THA are not limited to the absence of one thyroid lobe; they may include thyroglossal duct cysts, ectopic thyroid, absent superior, and inferior thyroid arteries, absent superior or recurrent laryngeal nerves, and absent parathyroid glands [22, 23]. Therefore, surgical procedures for THA combined with thyroid carcinoma should be more cautious: 1. Before surgery, doctors should understand the patient’s thyroid anatomy, including the location of the missing thyroid lobe and surrounding tissues. 2. Avoid injury to surrounding tissues, such as parathyroid tissue and recurrent laryngeal nerves. 3. Beware of ectopic thyroid tissue: THA patients may have ectopic thyroid tissue, which should be identified during surgery to avoid overlooking it. 4. THA with an isthmus should be resected together.

The diagnostic and therapeutic decisions in this case were also guided by the National guidelines for diagnosis and treatment of thyroid cancer 2022 in China (English version) [24], which provides recommendations consistent with international practice. Early detection and surgical removal of thyroid carcinoma generally confer an excellent prognosis, and the management of such patients often requires a multidisciplinary team (MDT) approach to optimize diagnostic evaluation, surgical planning, and postoperative surveillance [25].

For this case, the patient was diagnosed intraoperatively with papillary carcinoma of the right thyroid lobe, and a right lobectomy with prophylactic right central lymph node dissection was performed. An intraoperative frozen section analysis of the right thyroid nodule was performed, and the pathological result confirmed papillary thyroid carcinoma, supporting the decision to proceed with right lobectomy and prophylactic right central neck dissection. Intraoperatively, absence of the left thyroid lobe and presence of the left thyroid artery were confirmed, and the left parathyroid gland was intact. To avoid unnecessary damage, the left recurrent laryngeal nerve was not explored. Postoperatively, the patient recovered well and was treated with levothyroxine tablets for long‐term use. The patient was followed up at 1, 3, 6 months, and every 6 months thereafter with serum thyroglobulin (Tg), anti‐thyroglobulin antibodies (TgAb), and TSH measurement, along with neck ultrasonography. At the 2‐year follow‐up, thyroid function remained stable (TSH within target range), the Tg level was low and TgAb was negative, and ultrasonography showed no structural evidence of disease recurrence.

## 4. Conclusion

This case highlights the clinical importance of recognizing THA as a rare congenital anomaly that may coexist with papillary thyroid carcinoma. The absence of one thyroid lobe alters cervical anatomy and increases the complexity of preoperative evaluation and surgical management. Thorough imaging assessment, intraoperative frozen section analysis, and individualized operative planning are essential to avoid misdiagnosis and ensure complete tumor removal. Given that papillary thyroid carcinoma frequently metastasizes to the central cervical lymph nodes, prophylactic central neck dissection may be considered in selected patients. Early detection and surgical intervention generally lead to excellent outcomes, and MDT collaboration plays a key role in optimizing the diagnosis and treatment of such uncommon presentations.

## Ethics Statement

This study was approved by the Ethics Committee of Liuzhou People’s Hospital (Approval Number: KY2024–104–01).

## Consent

Written informed consent for publication of this case report and any accompanying images was obtained from the patient.

## Disclosure

All authors have read and approved the final manuscript, and agree to be accountable for all aspects of the work.

## Conflicts of Interest

The authors declare no conflicts of interest.

## Author Contributions

Conceptualization: Shaohua Chen, Yan Chen. Methodology: Shaohua Chen, Fengwei Wang. Investigation: Shenli Huang, Shuting Qin, Junyang Mo. Data curation and formal analysis: Fengwei Wang and Minmin Zhang. Writing – original draft: Shaohua Chen and Fengwei Wang. Writing – review and editing and supervision: Yan Chen. Shaohua Chen and Fengwei Wang contributed equally to this work.

## Funding

This work was supported by the Clinical Key Specialty (Department of Surgical Oncology) of Guangxi Zhuang Autonomous Region, the Liuzhou Science and Technology Plan Project (Grant 2024YB0101A009), and the Research Start‐up Fund of Liuzhou People’s Hospital (Grant LRYGCC202306).

## Data Availability

The data that support the findings of this study are available from the corresponding author upon reasonable request. The data are not publicly available due to privacy and ethical restrictions.
